# Triclocarban Disrupts the Epigenetic Status of Neuronal Cells and Induces AHR/CAR-Mediated Apoptosis

**DOI:** 10.1007/s12035-018-1285-4

**Published:** 2018-08-10

**Authors:** M. Kajta, A. Wnuk, J. Rzemieniec, W. Lason, M. Mackowiak, E. Chwastek, M. Staniszewska, I. Nehring, A. K. Wojtowicz

**Affiliations:** 1 Institute of Pharmacology, Department of Experimental Neuroendocrinology, Polish Academy of Sciences, Smetna Street 12, 31-343 Krakow, Poland; 2 Institute of Pharmacology, Department of Pharmacology, Laboratory of Brain Biostructure, Polish Academy of Sciences, Smetna Street 12, 31-343 Krakow, Poland; 3 Department of Cell Biology and Imaging, Confocal Microscopy Laboratory, Institute of Zoology, Jagiellonian University, Gronostajowa Street 9, 30-387 Krakow, Poland; 4 Institute of Oceanography, University of Gdansk, Al. Marszałka Piłsudskiego 46, 81-378 Gdynia, Poland; 5 Department of Animal Biotechnology, Faculty of Animal Sciences, University of Agriculture, Redzina Street 1B, 30-248 Krakow, Poland

**Keywords:** Apoptosis, DNA methylation, Primary neurons, Sumoylation, Triclocarban

## Abstract

Triclocarban is a phenyl ether that has recently been classified as a contaminant of emerging concern. Evidence shows that triclocarban is present in human tissues, but little is known about the impact of triclocarban on the nervous system, particularly at early developmental stages. This study demonstrated that triclocarban that was used at environmentally relevant concentrations induced apoptosis in mouse embryonic neurons, inhibited sumoylation, and changed the epigenetic status, as evidenced by impaired activities of HDAC, sirtuins, and DNMT, global DNA hypomethylation, and alterations of methylation levels of *bax*, *bcl2*, *Ahr*, and *Car* genes. The use of selective antagonists and specific siRNAs, which was followed by the co-localization of aryl hydrocarbon receptor (AHR) and constitutive androstane receptor (CAR) in mouse neurons, points to the involvement of AHR and CAR in triclocarban-induced neurotoxicity. A 24-h treatment with triclocarban enhanced protein levels of the receptors which was paralleled by *Car* hypomethylation and *Ahr* hypermethylation. *Car* hypomethylation is in line with global DNA hypomethylation and explains the increased mRNA and protein levels of CAR in response to triclocarban. *Ahr* hypermethylation could reflect reduced *Ahr* mRNA expression and corresponds to lowered protein levels after 3- and 6-h exposures to triclocarban that is likely related to proteasomal degradation of activated AHR. We hypothesize that the triclocarban-induced apoptosis in mouse neurons and the disruption of epigenetic status involve both AHR- and CAR-mediated effects, which may substantiate a fetal basis of the adult onset of neurological diseases; however, the expression of the receptors is regulated in different ways.

## Introduction

Triclocarban (3,4,4′-trichlorocarbanilide) is a phenyl ether that is often added to personal and health care products, including cloths, plastics, and even products dedicated for newborns, as well as pharmaceuticals. Long-term use of products containing triclocarban may enhance microbe-resistance against antibiotics used to fight infections [1]. Recent studies provided evidence that triclocarban is among the 10 most common pollutants of surface water. Its concentration and toxicity are higher than those attributed to triclosan [2]. In a recent ranking of potentially dangerous organic pollutants, antimicrobial compound, such as triclocarban, was positioned in the middle of the ranking, but with a higher ranking than bisphenol A (BPA), which has been recognized as a developmental risk factor. Triclocarban accumulates in human tissues, including the maternal and umbilical cord sera where it reaches the values of 2.75 and 0.82 μg/l, respectively [3]. This antimicrobial agent has been detected in urine and cord blood of mother/child pairs from Brooklyn, New York, as well as in two distinct brain regions of the human brain, i.e., the hypothalamus and white-matter tissue [4, 5]. In human brain, maximal concentration of triclocarban has been estimated at approximately 6 μg/kg. In rats, triclocarban provided by lactating females appeared to be lethal for the pups [6]. Nanomolar concentrations of triclocarban stimulated a cytotoxic effect of hydrogen peroxide in rat thymocytes, induced cancerogenesis, and altered the expression of thyroid hormone-regulated genes as well as the genes involved in lipid metabolism [7–10].

Although there is an increasing body of evidence on the deleterious effects of triclocarban, the systematic and complex data concerning the mechanisms of actions of triclocarban in neuronal cells are missing. Antimicrobial agents added as preservatives in hygiene and cosmetics commercial products have received particular attention. This is because epidemiological data showed strong correlations between exposures to environmental pollutants and increased risks of disorders, including neuropsychiatric and neurodegenerative diseases. Apoptosis (“self-killing”) is involved in neural development and the response of the nervous system to a variety of insults by cell elimination which includes the pathogenesis of neural degeneration, such as stroke, Huntington’s, Parkinson’s, and Alzheimer’s diseases [11]. There is, however, no report showing that triclocarban induces apoptosis in mammalian neurons. In addition, it is not clear whether triclocarban causes hypo- or hypermethylation of DNA and to what extent it alters the activities of histone deacetylases (HDACs), including sirtuins, histone acetyltransferases (HATs), or DNA methyltransferases (DMNTs). The recognition of these mechanisms is particularly important because triclocarban, through the alteration of epigenetic status and the dysregulation of apoptosis could impair neural development and/or cause neurodegenerations. Furthermore, the interactions of triclocarban with the aryl hydrocarbon receptor (AHR) and constitutive androstane receptor (CAR) signaling pathways at early developmental stages could cause abnormalities that may be revealed in adolescent or adult nervous systems and underlie the fetal basis of the adult onset of disease. The actions of triclocarban on AHR/CAR signaling could also involve posttranslational protein modifications. Numerous transcription regulators and nuclear receptors, including AHR, have been reported to be subjected to sumoylation that involves the covalent attachment of small ubiquitin-related modifier (SUMO) to specific lysine residue of the protein substrate [12].

Nuclear receptors such as the AHR and CAR have been shown to be the mediators of xenobiotic-induced changes [11, 13]. Until recently, AHR was almost exclusively considered to be responsible for dioxin and dioxin-like polychlorinated biphenyl (PCB) intoxications. However, it has become evident that AHR may also participate in neural development, since neural progenitor cells (NPCs) express robust levels of AHR and deletion of AHR disturbs neurogenesis and memory function in adult mice [14]. Interestingly, the AhR-knockout (AhR-KO) adult mice display a spontaneous horizontal nystagmus [15]. Previously, we demonstrated that AHR was involved in the propagation of pro-apoptotic action of dichlorodiphenyltrichloroethane (DDT), triclosan, and hypoxia [16–18]. CAR that is located in murine brain capillaries has been recognized as a xenobiotic-sensor which together with pregnane X receptor (PXR) can upregulate the functional expression of drug transporters, such as P-glycoprotein [19, 20]. Chronic uranium poisoning has been linked to CAR in the rat [21]. Our previous data demonstrated the presence of CAR in mouse neurons undergoing nonylphenol-induced apoptosis [22]. Recently, a novel mechanism that involves AHR and CAR signaling in di-(2-ethylhexyl)-phthalate-induced cerebellar toxicity has been recognized [23].

This study was aimed at investigating the mechanisms of action of triclocarban in mouse neurons in primary cultures, with a particular focus on the epigenetic statuses of neuronal cells, apoptosis as well as AHR- and CAR-mediated signaling. The authors assumed that the triclocarban-induced neurotoxicity involves AHR/CAR-mediated apoptosis and the disruption of the epigenetic status of neuronal cells. The apoptotic processes were determined after the assessment of the mitochondrial membrane potential, caspase-3 activity, BCL2/BAX ratio, and apoptotic fragmentation of cell nuclei. These were followed by measurements of reactive oxygen species (ROS) formation, lactate dehydrogenase (LDH) release, and neuronal cell death. The involvement of the AHR and CAR in triclocarban actions was verified using selective receptor antagonists and specific siRNAs. The levels of specific mRNAs and proteins were measured with quantitative polymerase chain reaction (qPCR), western blots (WBs), and ELISAs, and the cellular distributions of the AHR and CAR were demonstrated with a confocal microscope. The assessments of epigenetic statuses were based on measurements of HDAC, sirtuin, HAT, and DNMT activities as well as on the estimation of methylation levels of global DNA and the DNA of specific genes, including *Ahr*, *Car*, *bcl2*, and *bax*. To assess posttranslational protein modifications which could affect protein function in response to triclocarban, the level of sumoylation has been measured.

## Materials and Methods

### Materials

The α-naphthoflavone, Ac-DEVD-*p*NA (*N*-acetyl-asp-glu-val-asp *p*-nitro-anilide), anti-β-actin antibody, BSA (bovine serum albumin), CH223191 (1-methyl-*N*-[2-methyl-4-[2-(2-methylphenyl) diazenyl]phenyl-1*H*-pyrazole-5-carboxamide), CINPA (ethyl [5-[(diethylamino)acetyl]-10,11-dihydro-5*H*-dibenz[*b*,*f*]azepin-3-yl]carbamate), DMSO (dimethyl sulfoxide), poly-ornithine, EDTA-free protease inhibitors, glucose, H2DCFDA, Histone Acetyltransferase Activity Fluorometric Assay Kit, Histone Deacetylase Assay Kit, Imprint Methylated DNA Quantification Kit, NaCl, octylphenoxypolyethoxyethanol (IGEPAL CA-630), poly-ornithine, SDS, sodium deoxycholate, triclocarban (3,4′,4′-trichlorocarbanilide), and Tween 20 were purchased from Sigma-Aldrich (St. Louis, MO, USA). The calcein acetoxymethyl (AM), and Hoechst 33342 were purchased from Molecular Probes (Eugene, OR, USA). The B27 and neurobasal medium were obtained from Gibco (Grand Island, NY, USA), and the Bradford reagent was obtained from Bio-Rad Laboratories (Munchen, Germany). The cDNA reverse transcription kit, RNAlater, TaqMan Gene Expression Master Mix and TaqMan probes for the specific receptor genes *Ahr* and *Car*, and the apoptosis-related genes *bax* and *bcl-2* were obtained from Life Technologies Applied Biosystems (Carlsbad, CA, USA). The culture plates were obtained from TPP Techno Plastic Products AG (Trasadingen, Switzerland), the JC-1 Assay Kit was obtained from Biotium Inc. (Hayward, CA, USA), and the PVDF membranes were received from Merck Millipore (Billerica, MA, USA). The Cy3-conjugated anti-goat IgG, and Cy5-conjugated anti-rabbit IgG were obtained from Jackson Immunoresearch Laboratories Inc. (West Grove, PA, USA), and the BM Chemiluminescence Blotting Substrate, Cytotoxicity Detection Kit, Lysis Buffer (4.5 M guanidine-HCl, 100 mM sodium phosphate, pH 6.6), and mRNA isolation Kit were obtained from Roche Diagnostics GmbH (Mannheim, Germany). The DNMT Activity/Inhibition Assay Ultra Kit was from Abnova (Taipei, Taiwan). The enzyme-linked immunosorbent assays (ELISAs) for BCL2, BAX, AHR, and CAR were from Shanghai Sunred Biological Technology Co. (Sunred, China) and from EIAAB Science Co., LTD (Wuhan, China). The EpiTect MethyLight PCR Kit and RNeasy mini kit were from Qiagen (Valencia, CA, USA). The EZ DNA Methylation-Gold™ Kit and Quick-gDNA™ MicroPrep Kit were from Zymo Research (Irvine, CA). The donkey anti-goat IgG, donkey anti-rabbit IgG, goat polyclonal anti-AHR antibody (sc-8088), rabbit polyclonal anti-AHR antibody (sc-5579), rabbit polyclonal anti-BAX antibody (sc-493), rabbit polyclonal anti-BCL-2 antibody (sc-492), rabbit polyclonal anti-CAR antibody (sc-50462), siRNA AHR (sc-72178), and siRNA CAR (sc-43663) were purchased from Santa Cruz Biotechnology Inc. (Santa Cruz, CA, USA). The interferrin was from PolyPlus Transfection (Illkirch, France) and the Sirtuin Activity Assay Kit was purchased from BioVision (Milpitas, CA, USA). All other chemicals were of either analytical or laboratory grade, and purchased from standard suppliers.

### Primary Neuronal Cell Cultures

The tissue for the primary cultures originated from the neocortices of Swiss mouse embryos (Charles River, Germany) at 15–17 days of gestation. The animal care followed official governmental guidelines and all efforts were made to minimize suffering and the number of animals used. All procedures were carried out in accordance with the National Institutes of Health Guidelines for the Care and Use of Laboratory Animals. The studies were approved by the Bioethics Commission in compliance with Polish Law (21 August 1997). The cells were cultured as previously described [16, 24]. They were suspended in estrogen-free neurobasal medium supplemented with B27 and plated at a density of 2.5 × 10^5^ cells per cm^2^ on poly-ornithine (0.01 mg/ml)-coated multi-well plates. The cultures were maintained at 37 °C in a humidified atmosphere containing 5% CO_2_ for 7 days in vitro (DIV) prior to experimentation. The amount of astrocytes, as determined by the content of intermediate filament protein GFAP (glial fibrillary acidic protein), did not exceed 10% for all cultures [25].

### Treatment

Primary neuronal cell cultures were exposed to triclocarban (0.1–100 μM) for 3, 6, or 24 h. The chosen concentrations of triclocarban are environmentally relevant because the chemical has been found in animal tissues at similar doses [26–28]. Triclocarban-induced AHR activation was examined using the receptor antagonists α-naphthoflavone (1 μM) and CH223191 1-methyl-*N*-[2-methyl-4-[2-(2-methylphenyl) diazenyl]phenyl-1*H*-pyrazole-5-carboxamide; 1 μM). A new AHR antagonist CH223191 has been employed because α-naphthoflavone may partially inhibit the aryl hydrocarbon hydroxylase CYP1A1. The involvement of CAR signaling in triclocarban-induced effects was verified with the receptor antagonist CINPA (ethyl [5-[(diethylamino)acetyl]-10,11-dihydro-5*H*-dibenz[*b*,*f*]azepin-3-yl]carbamate; 1 μM). The α-naphthoflavone, CH223191, and CINPA were added to the culture media 45–60 min before triclocarban was added. To avoid non-specific effects in our study, specific receptor ligands were used at concentrations that did not affect the control levels of caspase-3 activity and LDH release (Table 1). All the compounds were originally dissolved in DMSO, whose concentration did not exceed 0.1%, and then further diluted in culture medium. The control cultures were treated with DMSO in concentrations equal to those used in the experimental groups.

**Table 1:** Effects of the AHR and CAR antagonists on the control spontaneous caspase-3 activity and LDH release in 7 DIV neocortical cultures. Primary neocortical cultures were treated with the AHR antagonists α-naphtoflavone and CH223191 and the CAR antagonist CINPA (all 1 μM) for 6 h. The results were normalized to the absorbency in vehicle-treated cells and expressed as a percentage of the control. Each value represents the mean of three independent experiments ± SEM. The number of replicates in each experiment ranged from 6 to 8

Neocortical cultures 6 h exposure	α-Naphtoflavone (1 μM)	CH223191 (1 μM)	CINPA (1 μM)
Caspase-3 activity [%control]	110 ± 14	112 ± 10	112 ± 14
LDH release [%control]	92 ± 12	89 ± 13	90 ± 14

### Assessment of Mitochondrial Membrane Potential

The JC-1 Assay Kit, which utilizes the cationic dye 5,5′,6,6′-tetrachloro-1,1′,3,3′-tetraethylbenzimidazolylcarbocyanine iodide has been used to determine the mitochondrial membrane potential as previously described [29–31]. In healthy cells, the dye aggregates and stains the mitochondria bright red, whereas in apoptotic cells, the mitochondrial membrane potential collapses, and the dye remains in the cytoplasm in a green fluorescent monomeric form [32]. The loss of mitochondrial membrane potential, which is a hallmark of apoptosis, was assessed in the neocortical cultures treated for 6 or 24 h with triclocarban. The cells were incubated with the JC-1 solution and then red (550 nm/600 nm) and green (485 nm/535 nm) fluorescence was measured with an Infinite M200PRO microplate reader (Tecan, Mannedorf, Switzerland). The data were analyzed with the i-control software, normalized to the fluorescence in the vehicle-treated cells, and expressed as the red to green fluorescence ratio ± SEM of three to four independent experiments. The fluorescence of the blanks, acting as no-enzyme controls, was subtracted from each value.

### Measurement of Caspase-3 Activity

The caspase-3 activity was measured according to [33], using samples treated for 6 h or 24 h with triclocarban alone or in combination with the test compounds as previously described [16, 34]. The cell lysates from neocortical cultures were incubated at 37 °C with the colorimetric substrate Ac-DEVD-*p*NA (*N*-acetyl-asp-glu-val-asp-*p*-nitro-anilide), which is preferentially cleaved by caspase-3. The amount of *p*-nitroanilide was monitored continuously for 60 min with a Infinite M200PRO microplate reader (Tecan, Mannedorf, Switzerland), and the results were analyzed with i-control software. The data from three to four independent experiments were normalized to the absorbency in the vehicle-treated cells and expressed as a percentage of the control ± SEM. The absorbency of the blanks, acting as no-enzyme controls, was subtracted from each value.

### Measurement of ROS Formation

In this study, the ability of triclocarban to induce reactive oxygen species (ROS) formation in the neocortical neurons was determined after application of 5 μM H2DCFDA. In general, H2DCFDA diffuses into the cells where it is deacetylated into a non-fluorescent compound that is subsequently oxidized by ROS into 2′,7′-dichlorofluorescein (DCF) [35, 36]. After a 40-min incubation, the culture medium was replaced with fresh Neurobasal, and the DCF fluorescence was measured at 3, 6, and 24 h during the experiment using an Infinite M200PRO microplate reader (Tecan, Mannedorf, Switzerland). The data were analyzed with the use of the Tecan I-control software and normalized to the fluorescence intensity in the vehicle-treated cells. The means ± SEM from eight separate samples were calculated from four independent experiments.

### Measurement of Lactate Dehydrogenase Activity

At 6 h or 24 h after the initial treatment with triclocarban, lactate dehydrogenase (LDH) that was released from damaged cells into the cell culture media was measured. The LDH release was estimated as previously described [18, 37]. The supernatants were collected from each well and incubated at room temperature for 30 to 60 min with the relevant reagent mixture according to the supplier’s instructions (Cytotoxicity Detection Kit). The intensity of the red color formed in the assay, measured at a wavelength of 490 nm, was proportional to both LDH activity and the number of damaged cells. The data from three to four independent experiments were normalized to the color intensity of the vehicle-treated cells (100%) and expressed as a percentage of control.

### Staining with Hoechst 33342 and Calcein AM

The apoptotic cells were stained with Hoechst 33342 after 24 h, as previously described [38]. The neocortical cells cultured on glass cover slips were washed with 10 mM phosphate-buffered saline (PBS) and exposed to the stain (0.6 mg/ml), at room temperature for 5 min. The cells with bright blue fragmented nuclei showing condensed chromatin were determined to be apoptotic cells. The staining with calcein AM utilized intracellular esterase activity in neocortical cells that were treated with 10 μM triclocarban for 24 h [34]. The cells that were grown on glass cover slips were washed with PBS and then incubated in 2 μM calcein AM in PBS at room temperature for 10 min. The cells with bright green cytoplasm were identified as living cells. A fluorescence microscope (NIKON Eclipse 80i, NIKON Instruments Inc., Melville, NY, USA) equipped with a camera with BCAM Viewer Basler AG software was used for analyses. Fluorescence intensity was monitored at Ex/Em 494/520 nm.

### Estimation of Histone Deacetylase Activities

#### HDAC Activity

The HDAC activity was detected by utilizing the Histone Deacetylase Assay Kit (Sigma-Aldrich, St. Louis, MO, USA) which contained HeLa cell lysate to be used as a positive control. According to the manufacturer’s instructions, the fluorescence measured at *λ*_ex_ = 365 nm/*λ*_em_ = 460 nm was proportional to the HDAC activity in the sample. The HDAC activity was measured at 24 h after the initial treatment.

#### Sirtuin Activity

The Sirtuin Activity Assay Kit (BioVision, Milpitas, CA, USA) was used to determine the sirtuin activity. In the presence of NAD^+^, sirtuins are able to generate the deacetylated p53-AFC substrate, nicotinamide, and O-acetyl-ADP ribose. The cleavage of the substrate releases the fluorescent group, which is detected fluorometrically at ex/em = 400/505 nm. Because the p53-AFC substrate can also be deacetylated by non-sirtuin HDACs, Trichostatin A is added to the reaction to specifically inhibit non-sirtuin activities in the samples. The manufacturer provided undefined sample to be used as a positive control. The sirtuin activity was measured at 24 h after the initial treatment.

### Estimation of Histone Acetyltransferase (HAT) Activity

The HAT activity was determined with the use of the Histone Acetyltransferase Activity Fluorometric Assay Kit (Sigma-Aldrich, St. Louis, MO, USA). In the assay, the generated product of histone acetyltransferase activity was detected fluorimetrically at *λ*_ex_ = 535/*λ*_em_ = 587 nm. The manufacturer provided an active nuclear extract to be used as a positive control. The HAT activity was measured at 24 h after the initial treatment.

### Estimation of DNA Methyltransferase (DNMT) Activity

The DNMT activity was measured using the DNMT Activity/Inhibition Assay Ultra Kit (Abnova, Taipei, Taiwan) that includes microplate wells coated by a universal DNMT substrate. In this assay, the DNMT enzymes transfer a methyl group to a cytosine to methylate DNA that can be recognized with an anti-5-methylcytosine antibody. The amount of methylated DNA is proportional to the enzymatic activity, and it can be measured through an ELISA-like reaction by reading the absorbance at a wavelength of 450 nm. MU4 DNMT Enzyme Control has been provided as a positive control with activity of both maintenance and de novo DNMT. The DNMT activity was assessed in 10 μg of nuclear extract per sample, at 6 h post-treatment.

### Analyses of DNA Methylation

#### Global DNA Methylation

To determine the methylation statuses of the DNA in the neocortical cells that were exposed to triclocarban, an Imprint Methylated DNA Quantification Kit was utilized. The capture and detection antibodies detected the methylated DNA that was quantified colorimetrically as previously described [38–40]. The purified DNAs were added to a 96-well plate (150 ng/well) where they bound with the capture antibody. The developing solution was added to monitor the reactions for color changes. The calculations of the relative global methylation levels were based on measurements of the absorbance at 450 nm. For each sample, the DNA quantity was determined spectrophotometrically (ND/1000 UV/Vis; Thermo Fisher NanoDrop, USA). The global DNA methylation was assessed in the samples which were collected at 6 h post-treatment.

#### DNA Methylation in Specific Genes

Genomic high-quality and ultra-pure DNA was extracted from neocortical cells using a Quick-gDNA™ MicroPrep Kit (Zymo Research, Irvine, CA) as previously described [39]. The quantity of DNA was spectrophotometrically determined at 260 nm and 260/280 nm (ND/1000 UV/Vis; Thermo Fisher NanoDrop, USA). Complete bisulfite conversion of GC-rich DNA was performed by the use of the EZ DNA Methylation-Gold™ Kit (Zymo Research, Irvine, CA). The kit integrates DNA denaturation and bisulfite conversion processes into one-step. The bisulfite-converted samples were eluted in a 10-μl volume and stored at − 80 °C until needed. The qPCR reaction (MethyLight) was developed using the EpiTect MethyLight PCR Kit (Qiagen, Valencia, CA). Sets of TaqMan probes, designed specifically for bisulfite-converted DNA sequences, were used: a set representing fully methylated and fully unmethylated probes for the *Ahr*, *Car*, *bcl2*, and *bax* promoters and the internal reference set for the *Hprt* gene to control for input DNA. In EpiTect MethyLight Assays, the methylation specific TaqMan probe contains FAM™ as 5′ reporter dye, whereas the unmethylation specific TaqMan probe is linked to VIC^®^. Measuring the release of FAM and VIC is used to determine the methylation status, whereby the ratio of measured Ct values with both fluorescence dyes allows quantification of the methylation. The assays enable the direct calculation of the methylation degree in a sample by taking the threshold cycles determined with each of both dyes: percentage of methylation [%]: C_meth_ = 100/[1 + 2^(ΔCt meth − ΔCt unmeth)^] according to [41]. The DNA methylation in specific genes was determined at 6 h post-treatment.

### Determination of Sumoylation

To measure the protein sumoylation, a Global Protein Sumoylation Kit (Abcam, Cambridge, UK) that is designed for measuring sumoylation of the targeted proteins was used. Sumoylation of the proteins was detected by recognition of SUMO conjugated to the proteins with an anti-SUMO antibody, and it was quantified through the absorbance signal report-color development system at 450 nm. The protein sumoylation was estimated at 24 h post-treatment.

### Silencing of AHR and CAR

The *Ahr* and *Car* siRNAs were used to destroy their mRNAs and inhibit protein expression in neocortical cells. Each siRNA was applied separately for 6 h at 50 nM in antibiotic-free medium containing interferrin. After transfection, the culture media were changed, and the cells were incubated for 12 h before starting the experiment. The controls included positive siRNAs (control) and a negative siRNA (negative control) containing a scrambled sequence that did not cause degradation of any known cellular mRNA. The effectiveness of the mRNA silencing was verified by measurement of specific mRNAs with qPCR. As previously, mRNA silencing resulted in approximately 70 and 40% decrease in mRNA concentrations for *Ahr* and *Car*, respectively [16, 22].

### Real-Time PCR Analysis of mRNAs Specific to Genes Coding the Receptors *Ahr* and *Car* and Apoptosis-Related *bcl2* and *bax*

In the present study, total RNA was extracted from neocortical cells at 7 days in vitro at 3, 6, and 24 h (approximately 1.5 × 10^6^ cells per sample) with a Qiagen RNeasy mini kit. The quantity of RNA was determined at 260 nm and 260/280 nm (ND/1000 UV/Vis; Thermo Fisher NanoDrop, USA). The two-step qPCR involved the reverse transcription (RT) reaction and qPCR which were run in the CFX96 Real-Time System (Bio-Rad, USA) as previously described [38]. The RT reaction was performed using the cDNA reverse transcription kit at a final volume of 20 μl with 300 ng of RNA as a cDNA template. Products of the RT reaction were amplified with the TaqMan Gene Expression Master Mix kit (Life Technologies Applied Biosystems, USA) using TaqMan probes as primers for the specific genes encoding the receptors *Ahr* and *Car* and the apoptosis-related *bcl2* and *bax*. The amplification was carried out in a mixture with a total volume of 20 μl containing 1× TaqMan Gene Expression Master Mix and 1 μl of RT product, which was used as the PCR template. The standard qPCR procedures were performed as follows: 2 min at 50 °C and 10 min at 95 °C followed by 40 cycles of 15 s at 95 °C and 1 min at 60 °C. The threshold value (Ct) for each sample was set during the exponential phase, and the delta delta Ct method was used for data analysis. *Hprt* (hypoxanthine phosphoribosyltransferase coding gene) was used as a reference gene.

### Western Blot Analysis

The cells that were exposed to triclocarban for 3, 6, or 24 h were lysed in ice-cold lysis buffer (50 mM Tris HCl, pH 7.5, 100 mM NaCl, 0.5% sodium deoxycholate, 0.5% octylphenoxypolyethoxyethanol (IGEPAL CA-630), EDTA-free protease inhibitors, and 0.5% SDS). The lysates were sonicated and centrifuged 15,000×*g* at 4 °C for 30 min and the protein concentrations in the supernatants were determined with the Bradford reagent (Bio-Rad Protein Assay) using BSA as a standard. The samples that contained 40 μg of total protein were reconstituted in sample buffer (125 mM Tris, pH 6.8, 4% SDS, 25% glycerol, 4 mM EDTA, 20 mM DTT, and 0.01% bromophenol blue), denatured, and separated on a 7.5% SDS-polyacrylamide gel using a Bio-Rad Mini-Protean II Electrophoresis Cell, as previously described [18, 39, 42]. After electrophoretic separation, the proteins were electrotransferred to PVDF membranes using the Bio-Rad Mini Trans-Blot apparatus. Next the membranes were washed, and non-specific binding sites were blocked with 5% dried milk and 0.2% Tween 20 in 0.02 M TBS (Tris-buffered saline) for 2 h. This was followed by a 12-h incubation (at 4 °C) with one of the following primary antibodies (Santa Cruz Biotechnology): anti-AHR rabbit polyclonal antibody (diluted 1:100), anti-CAR rabbit polyclonal antibody (diluted 1:50), anti-BCL2 rabbit polyclonal antibody (diluted 1:100), anti-BAX rabbit polyclonal antibody (diluted 1:150), or anti-β-actin mouse monoclonal antibody (diluted 1:3000) diluted in TBS/Tween. The membranes were then washed and incubated for 2 h with horseradish peroxidase-conjugated IgG (donkey anti-goat or goat anti-rabbit IgG, Santa Cruz Biotechnology) diluted at 1:1000 in TBS/Tween 20. To control for the amount of denatured protein that was loaded onto the gel, the membranes were stripped and reprobed with an anti-β-actin antibody (Sigma-Aldrich). The signals were developed by chemiluminescence (ECL) using the BM Chemiluminescence Blotting Substrate (Roche Diagnostics GmbH) and visualized with the Luminescent Image Analyzer Fuji-Las 4000 (Fuji, Japan). The immunoreactive bands were quantified by an image analyzer (ScienceLab, MultiGauge V3.0).

### Enzyme-Linked Immunosorbent Assays

The levels of the receptors AHR and CAR as well as apoptosis-related BCL2 and BAX were determined in neocortical cells 24 h after treatment with triclocarban. Specific detection was obtained by enzyme-linked immunosorbent assays (ELISAs) and the use of the quantitative sandwich enzyme immunoassay technique. The monoclonal antibodies specific for AHR, CAR, BCL2, or BAX were pre-coated onto a 96-well plate. The standards and non-denatured cell extracts were added to the wells, which resulted in the capturing of all native proteins by the immobilized biotin-conjugated antibodies. Following a wash to remove any unbound substances, horseradish peroxidase-conjugated avidin was added to bind the biotin. After another wash and adding a substrate solution, the enzyme reaction yielded a blue product. The absorbance was measured at 450 nm and was proportional to the amounts of specific proteins. The total protein concentration was determined in each sample with the Bradford reagent (Bio-Rad Protein Assay).

### Immunofluorescent Labeling for AHR, CAR, and MAP-2 and Confocal Microscopy

To determine the cellular localization of AHR and CAR and to confirm the neuronal nature of the neocortical cells, the cells were grown on glass cover slips and subjected to immunofluorescence double-labeling at 7 DIV, as previously described [16, 29]. After a 1-h incubation in blocking buffer (5% normal donkey serum and 0.3% Triton X-100 in 0.01 M PBS), the cells were treated for 24 h with primary antibodies: anti-AHR goat polyclonal (1:50), anti-CAR rabbit polyclonal (1:50), and anti-MAP-2 rabbit polyclonal (1:100). This step was followed by a 24-h incubation in a mixture of secondary antibodies including Cy3-conjugated anti-goat IgG (1:300) and Cy5-conjugated anti-rabbit IgG (1:300). The samples were then washed, mounted, and covered with a cover-slip. They were analyzed with a confocal laser scanning microscope Zeiss LSM 510 META (Carl Zeiss MicroImaging GmbH, Jena, Germany) using a Plan-Neofluar 40×/1.3 Oil DIC objective. A He/Ne laser and an argon laser, with two laser lines emitting at 514 and 647 nm, were used to excite the Cy3- and Cy5-conjugated antibodies, respectively. The fluorescence signal was enhanced by summing four scans per line. A pinhole value of 1 airy unit was used to obtain flat images. The confocal microscopy data were quantified by an ImageJ software and the corrected total cell fluorescence (CTCF) was calculated according to the formula: CTCF = integrated density − (area of selected cell × mean fluorescence of background readings).

### Data Analysis

The statistical tests were performed on raw data expressed as the mean arbitrary absorbance or fluorescence units per well containing 50,000 cells for the caspase-3, LDH, mitochondrial membrane potential, and ROS formation assays, as the absorbance units per 10 μg of nuclear extract for DNMT activity, as the fluorescence units per 1.5 million cells for qPCR, as the mean optical density per 40 μg of protein for western blot assays, as the pg of AHR, CAR, BCL2, and BAX per μg of total protein for the ELISAs, as the ng per μl of protein for the sumoylation assay, as the ng of methylated DNA per 150 ng (1 μl) of DNA sample for global DNA methylation assay, as nmol per μg of protein sample for HAT activity, as pM for the sirtuin activity, or as μM for the HDAC activity. A one-way analysis of variance (ANOVA) was preceded by the Levene test of homogeneity of variances and was used to determine overall significance. The differences between the control and experimental groups were assessed with a post hoc Newman-Keuls test. Significant differences were marked in the following ways: *p < 0.05, **p < 0.01, and ***p < 0.001 (versus control cultures) and ^#^*p* < 0.05, ^##^*p* < 0.01, and ^###^*p* < 0.001 (versus the cultures exposed to triclocarban). The results were expressed as the mean ± SEM of three to four independent experiments. The number of replicates ranged from 6 to 8, except for the measurements of sirtuin activity, CTCF, and western blot analyses, whose number of replicates ranged from 2 to 5. To compare the effects of triclocarban in different treatment paradigms, the results corresponding to caspase-3, LDH, mitochondrial membrane potential, ROS formation, DNMT, and western blot analyses were presented as a percentage of the control.

## Results

### Effects of Triclocarban on Mitochondrial Membrane Potential and ROS Formation

#### Triclocarban Led to a Loss of Mitochondrial Membrane Potential

**Fig. 1:**
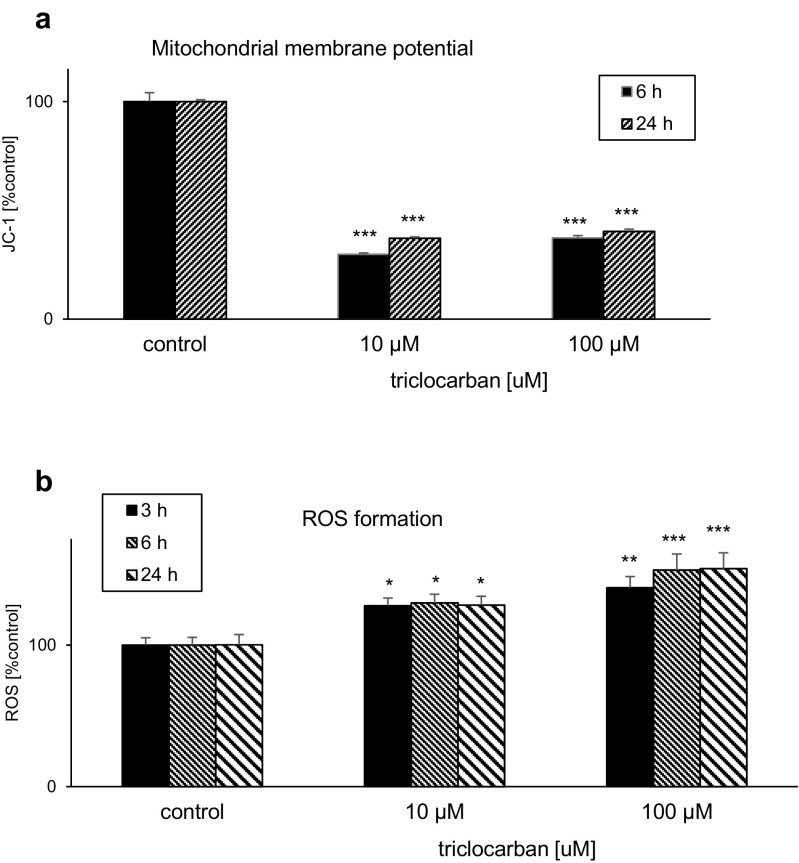
Triclocarban (10 and 100 μM) led to a loss of mitochondrial membrane potential (**a**) and stimulated ROS formation (**b**) in primary cultures of mouse neocortical cells. The cells were treated with triclocarban for 3, 6, or 24 h. The results are presented as a percentage of the control. Each bar represents the mean ± SEM. The number of replicates in each experiment ranged from 6 to 8. *p < 0.05, **p < 0.01, and ***p < 0.001 versus the control cultures

Triclocarban used at concentrations 10 or 100 μM reduced the mitochondrial membrane potential in neocortical cultures. The effects depended on the concentration and the duration of exposure and they were most pronounced at 6 h during the experiment (Fig. 1a). This effect supports the ability of triclocarban to activate the mitochondrial apoptotic pathway in mouse neurons.

#### Triclocarban Stimulated ROS Formation

In neocortical cultures exposed to 10 or 100 μM triclocarban, the ROS formation increased to 128–140% at 3 h and 130–153% at 6 h. The effects of triclocarban were concentration and time dependent. The ROS formation remained enhanced until 24 h when triclocarban stimulated ROS production to the level of 128–154% of the control (Fig. 1b).

### Effects of Triclocarban on mRNA and Protein Levels of BCL2 and BAX in Neocortical Cultures

#### Triclocarban Decreased the *bcl2*/*bax* Ratio

**Fig. 2:**
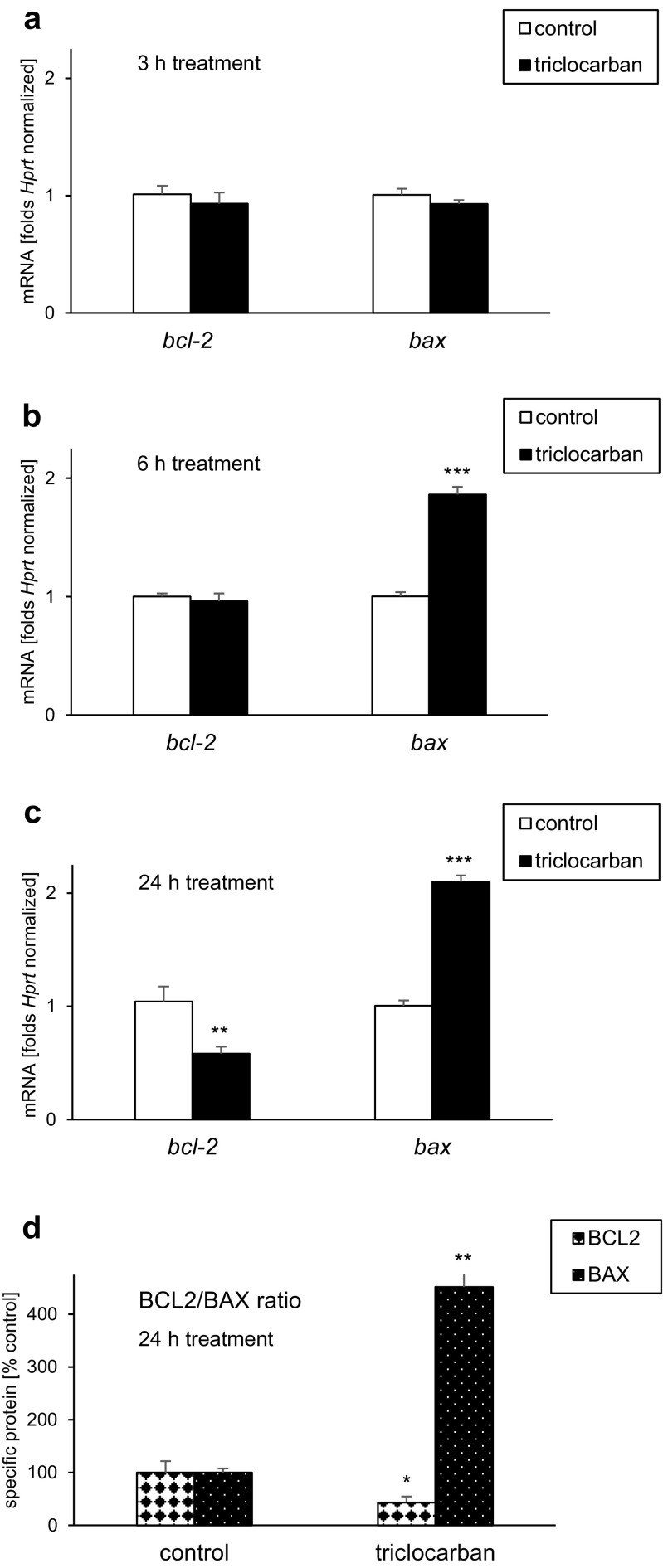
Triclocarban (10 μM) decreased the *bcl2*/*bax* mRNA ratio (**a**–**c**) and the BCL2/BAX protein ratio (**d**) in primary cultures of mouse neocortical cells. The cells were treated with triclocarban for 3, 6, or 24 h. The results are presented as folds, which were *Hprt* normalized, or a percentage of the control. Each bar represents the mean ± SEM. The number of replicates in each experiment ranged from 6 to 7. **p < 0.01 and ***p < 0.001 versus the control cultures

Neocortical cultures exposed for 3 h to triclocarban (10 μM) expressed approximately as much *bcl2* and *bax* mRNAs as the control cultures (Fig. 2a). However, a 6-h treatment with triclocarban resulted in a 86% increase in the level of *bax* mRNA (Fig. 2b). A 24-h treatment with triclocarban lead to approximately 50% decrease in the level of *bcl2* mRNA that was accompanied by over twofold increase in the level of *bax* mRNA (Fig. 2c). In the cells exposed for 6 or 24 h to triclocarban, the *bcl2*/*bax* ratio was diminished compared to the control cells.

#### Triclocarban Decreased the BCL2/BAX Ratio

The specific ELISAs revealed that a 24-h treatment with 10 μM triclocarban reduced the level of anti-apoptotic BCL2 approximately 57% from 12 to 5 pg/μg of total protein (Fig. 2d). In contrast to BCL2, the pro-apoptotic BAX protein level increased from 0.001 to 0.005 pg/μg in response to triclocarban, which is equal to a fivefold enhancement. Therefore, triclocarban diminished the BCL2/BAX ratio, which is a feature of apoptosis, and in particular, of the mitochondrial pathway.

### Effects of Triclocarban on Hoechst 33342 and Calcein AM Stainings in Hippocampal Cultures

**Fig. 3:**
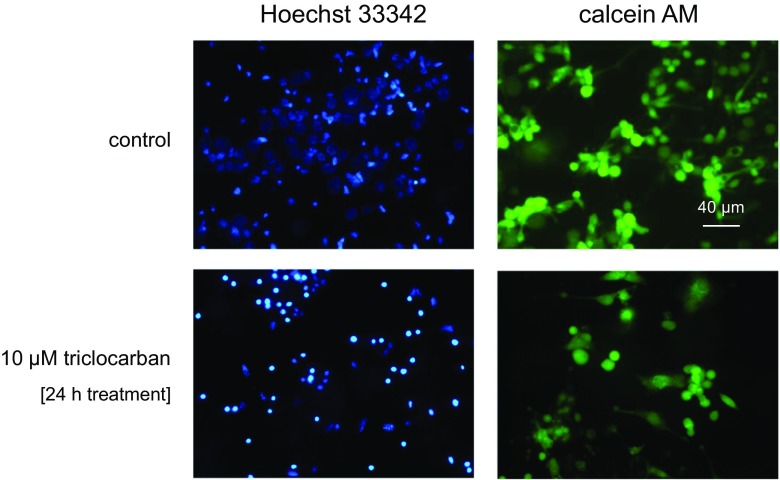
The effects of triclocarban (10 μM) on Hoechst 33342 (1st column) and calcein AM (2nd column) staining in neocortical cultures, examined 24 h post-treatment. The cells with bright, fragmented nuclei showing condensed chromatin were identified as undergoing apoptosis, whereas cells with light-colored cytoplasm were identified as living cells

In this study, a 24-h exposure of neocortical cultures to triclocarban (10 μM) enhanced the number of Hoechst 33342-stained nuclei that displayed condensed chromatin, which is a feature of apoptosis (Fig. 3). Moreover, the treatment with triclocarban reduced the density of calcein AM-stained cells that exhibit a light-colored cytoplasm, which is typical of living cells.

### Effects of Triclocarban and Selective Receptor Antagonists on Caspase-3 Activity and LDH Release

#### Triclocarban Induced Caspase-3 Activity and LDH Release

**Fig. 4:**
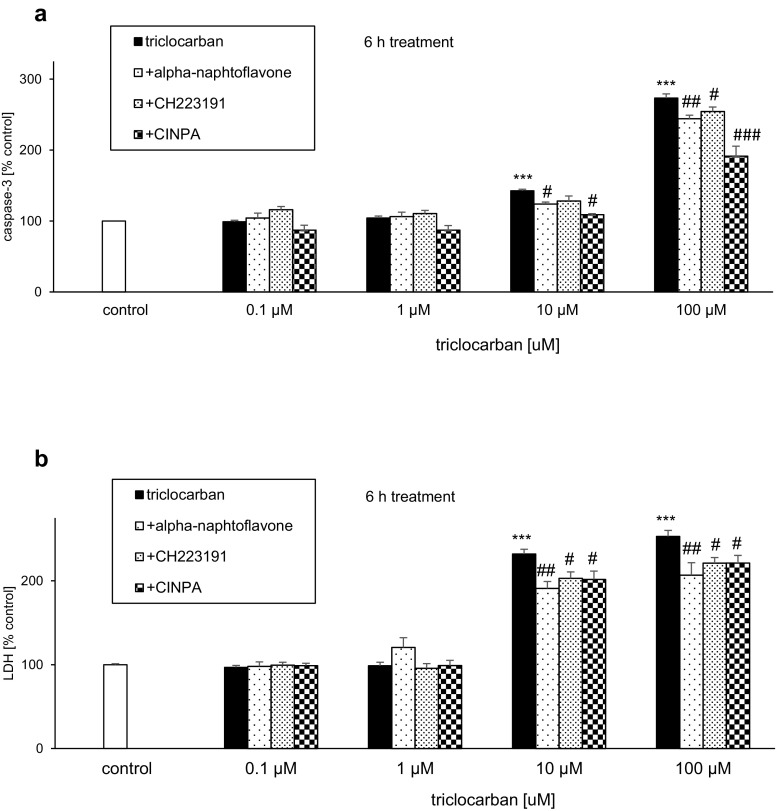
Triclocarban (10 and 100 μM) induced caspase-3 activity (**a**) and LDH release (**b**) in neocortical cell cultures, and selective AHR and CAR antagonists reduced these effects. The primary neocortical cultures were treated with triclocarban (0.1, 1, 10, and 100 μM) for 6 h. The AHR antagonists α-naphthoflavone and CH223191 and the CAR antagonist CINPA (all 1 μM) were added into the culture media approximately 45–60 min before triclocarban was added. The results are presented as a percentage of the control. Each bar represents the mean ± SEM. The number of replicates in each experiment ranged from 6 to 8. ***p < 0.001 versus the control cultures; ^#^*p* < 0.05, ^##^*p* < 0.01, and ^###^*p* < 0.001 versus the cultures exposed to triclocarban

In neocortical cultures that were treated with triclocarban (0.1–100 μM) for 6 h, the level of caspase-3 activity ranged from 143 to 273% of the control value (Fig. 4a). The triclocarban-evoked LDH release was enhanced to 232–253% of the control level (Fig. 4b). In the study, the only effective concentrations of triclocarban were 10 and 100 μM.

#### Selective AHR and CAR Antagonists Reduced the Effects of Triclocarban

The AHR antagonists α-naphthoflavone and CH223191 (both 1 μM) inhibited the triclocarban (10 and 100 μM)-induced caspase-3 activity by 14–29% at 6 h (Fig. 4a). These effects were accompanied by a significant 29–46% inhibition of triclocarban-evoked LDH release in the presence of α-naphthoflavone and CH223191 (Fig. 4b).

Compared with the effects of the AHR antagonists on triclocarban-induced caspase-3 activity, the effect the CAR antagonist CINPA (1 μM) was more pronounced since it decreased the triclocarban-stimulated enzyme activity by 34–82% (Fig. 4a). However, CINPA diminished the triclocarban-induced LDH release by approximately 30%, which is similar to the effects of α-naphthoflavone and CH223191 (Fig. 4b).

### AHR and CAR siRNAs Limited the Effects of Triclocarban in Neocortical Cell Cultures

#### AHR siRNA-Transfected Cells

**Fig. 5:**
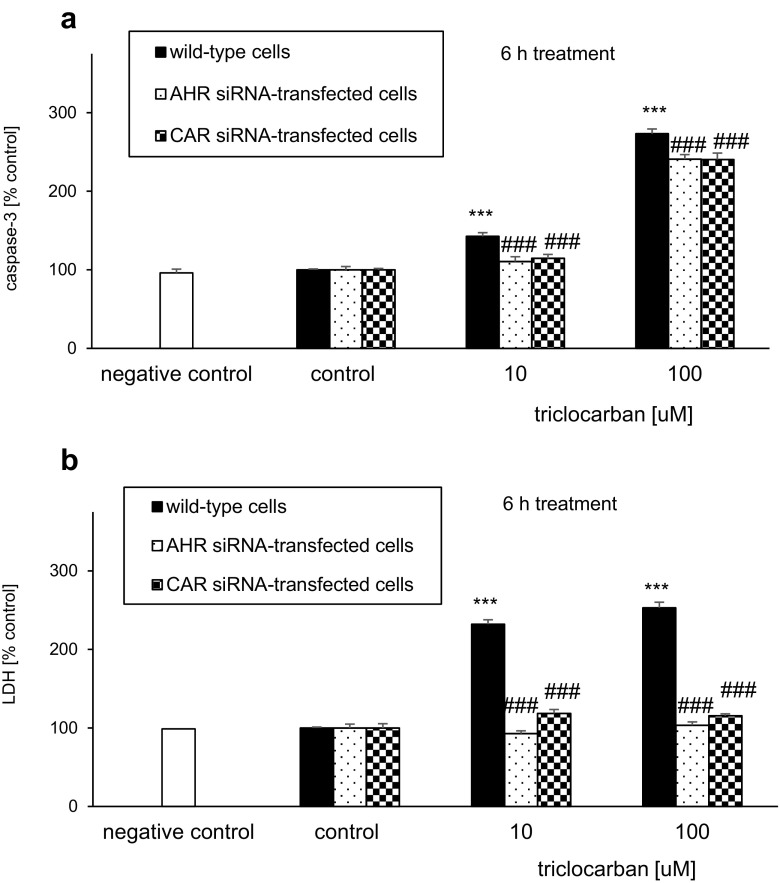
AHR and CAR siRNAs limited the triclocarban (10 and 100 μM)-induced caspase-3 activity (**a**) and LDH release (**b**) in neocortical cell cultures, examined 6 h post-treatment. The primary neocortical cultures were transfected with 50 nM siRNA in interferrin-containing medium without antibiotics for 6 h. In addition, a negative siRNA containing a scrambled sequence that did not lead to the specific degradation of any known cellular mRNA was included. The results were normalized to the absorbency in vehicle-treated cells and expressed as a percentage of the control. Each bar represents the mean ± SEM. The number of replicates in each experiment ranged from 6 to 8. ***p < 0.001 versus the wild-type control cultures; ^###^*p* < 0.001 versus the wild-type cultures exposed to triclocarban

A 6-h exposure to triclocarban (10 and 100 μM) induced caspase-3 activity and LDH release in AHR siRNA-transfected cells (Fig. 5). The transfected cells were less vulnerable to triclocarban-induced effects and reached values 32% (caspase-3 activity) and 139–150% (LDH release) lower than the non-siRNA-treated wild-type cells.

#### CAR siRNA-Transfected Cells

Triclocarban (10 and 100 μM) activated caspase-3 and caused LDH release in CAR siRNA-transfected cells at 6 h of treatment. However, the level of caspase-3 activity was 28–33% lower in CAR siRNA-transfected cells than in non-transfected wild-type cells (Fig. 5a). With respect to LDH, the effect of triclocarban was 114–137% lower in CAR siRNA-transfected cells than in non-transfected wild-type cells (Fig. 5b). The results suggest a reduced vulnerability of the siRNA-transfected cells to the apoptotic actions of the compound.

### mRNA Expression Levels of AHR and CAR in Triclocarban-Treated Neocortical Cultures

**Fig. 6:**
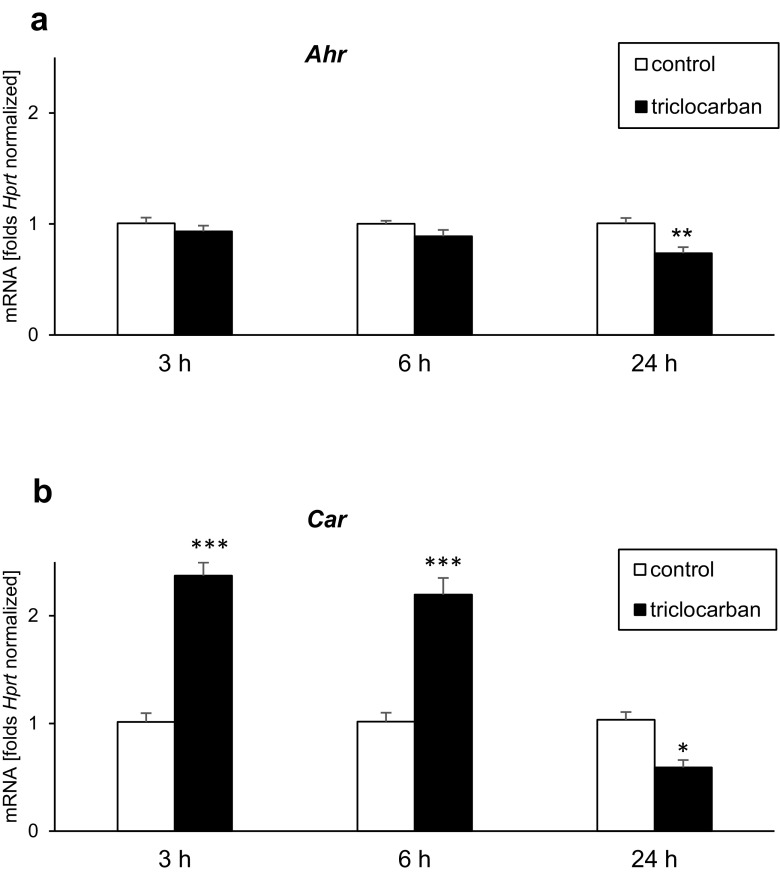
Triclocarban (10 μM) inhibited *Ahr* mRNA expression (**a**) and altered expression levels of *Car* mRNA (**b**). The primary neocortical cultures were treated with triclocarban for 3 to 24 h. Extraction of total RNA was followed by reverse transcription (RT) and qPCR. The products of the RT reaction were amplified using TaqMan probes and primers for the specific genes. *Hprt* was used as a reference gene. Each bar represents the mean ± SEM. The number of replicates ranged from 6 to 8. *p < 0.05, **p < 0.01, and ***p < 0.001 versus the control cultures

Neocortical cultures exposed for 3 or 6 h to triclocarban (10 μM) expressed approximately as much *Ahr* mRNA as the control cultures (Fig. 6a). However, a 24-h exposure to triclocarban resulted in a 27% decrease in the level of *Ahr* mRNA.

Treatment of neocortical cultures with triclocarban (10 μM) for 3 to 6 h caused over a twofold increase in *Car* mRNA expression compared with the control cultures (Fig. 6b). A prolonged 24-h exposure to triclocarban caused a 43% decrease in the level of *Car* mRNA.

### Protein Levels of AHR and CAR in Triclocarban-Treated Neocortical Cultures

#### AHR and CAR Protein Levels Measured by ELISAs

**Fig. 7:**
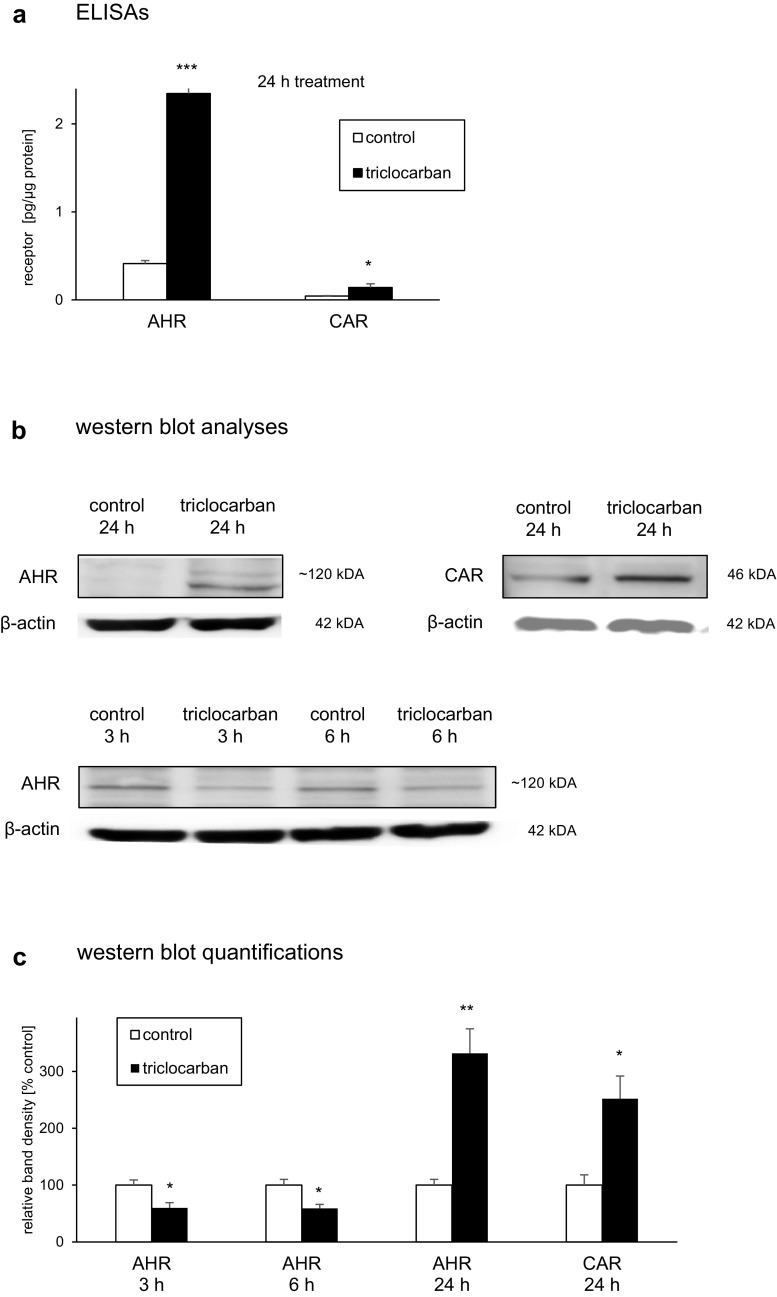
Triclocarban (10 μM) increased the protein level of CAR and altered protein levels of AHR that were measured by ELISAs (**a**) or analyzed by western blots (**b**, **c**). The primary neocortical cultures were treated with triclocarban for 3 to 24 h. The collected protein samples were assayed with the use of specific ELISAs or denatured, electrophoretically separated, transferred to PVDF membrane, and subjected to immunolabeling. The signals were developed by chemiluminescence (ECL) and visualized with Luminescent Image Analyzer Fuji-Las 4000 (Fuji, Japan). The immunoreactive bands were quantified by an image analyzer (ScienceLab, MultiGauge V3.0) and the relative protein levels of the AHR and CAR were presented as a percentage of the control. Each bar represents the mean ± SEM. The number of replicates ranged from 2 to 3 (western blots) or 6 to 8 (ELISAs). *p < 0.05, **p < 0.01, and ***p < 0.001 versus the control cultures

In the control neocortical cultures, the levels of AHR and CAR reached values of 0.4 and 0.04 pg/μg of total protein, respectively (Fig. 7a). A 24-h exposure to triclocarban (10 μM) resulted in 1.9 pg/μg increase in the level of AHR and 0.1 pg/μg increase in the protein expression of CAR.

#### AHR and CAR Protein Levels Analyzed by Western Blot

The western blot analyses demonstrated the constitutive protein expression of the AHR and CAR in mouse neocortical cells (Fig. 7b). The immunoblottings revealed that a prolonged 24-h exposure of neocortical cultures to triclocarban (10 μM) stimulated AHR and CAR protein expression to 332% and 252% of the control level, respectively (Fig. 7c). However, short term 3- and 6-h exposures to triclocarban inhibited AHR protein expression by approximately 40%, possibly due to proteasomal degradation of the activated receptor.

### Cellular Distribution of AHR and CAR in Neocortical Cultures Treated with Triclocarban—a Confocal Microscopic Analysis

**Fig. 8:**
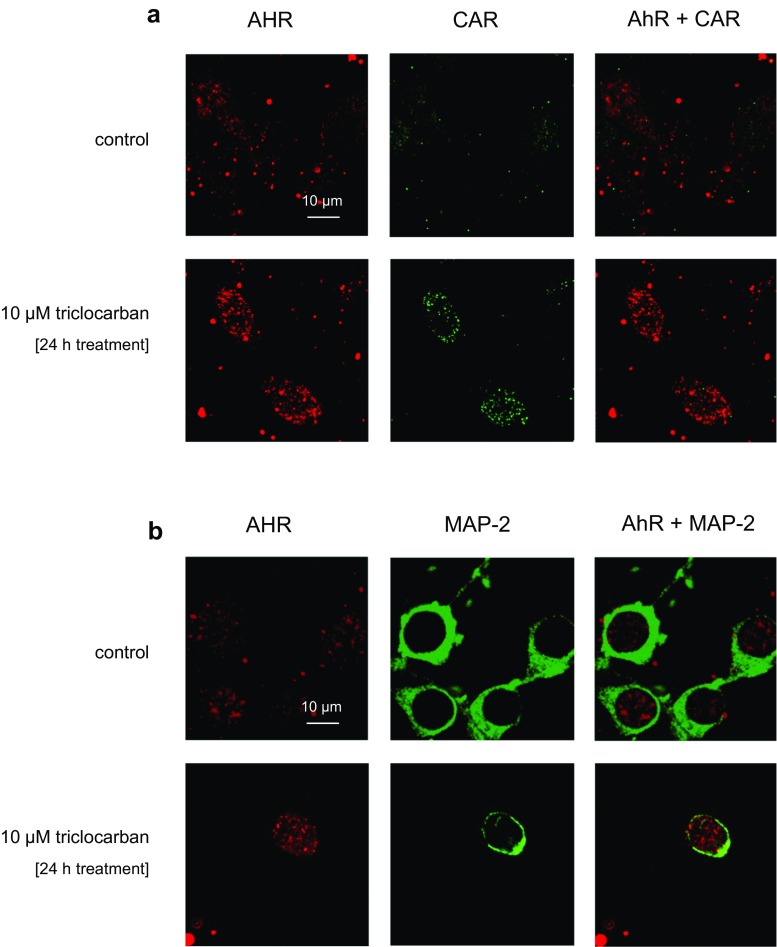
The influence of triclocarban (10 μM) on the cellular distribution of AHR (red) and CAR (green) in mouse neocortical cultures, examined 24 h after the initial treatment. The overlay of AHR and CAR (green plus red) and MAP-2 (green) immunostaining images are also shown. The cells were cultured on glass cover slips and subjected to immunofluorescent double labeling. The samples were analyzed with a confocal laser scanning spectral microscope LSM510 META, Axiovert 200 M (Carl Zeiss MicroImaging GmbH, Jena, Germany) using a Plan-Apochromat 63×/1.4 Oil DIC objective

The immunofluorescence labeling of the receptors was performed in parallel with the measurements of their protein levels by ELISAs or western blots. Confocal microscopy showed that the AHR and CAR were localized in neocortical cells, where the cells had red and green immunofluorescence, respectively (Fig. 8a, b). A 24-h exposure to triclocarban (10 μM) significantly increased the AHR and CAR staining. For AHR and CAR, the CTCF values increased from 32,626 and 7282 to 177,714 and 66,057, respectively (Table 2). The neuronal nature of the cells was confirmed by MAP-2 immunostaining, in the AHR-positive cells. Furthermore, the MAP-2 immunostaining provided evidence of triclocarban-evoked inhibition of neurite outgrowth.

**Table 2:** The influence of triclocarban (10 μM) on the cellular expression of AHR and CAR in mouse neocortical cultures, examined 24 h after the initial treatment. The confocal microscopy data were quantified by the corrected total cell fluorescence (CTCF). The number of replicates in each experiment was 5

Corrected total cell fluorescence 24 h exposure	AHR	CAR
Control	32,626 ± 8442	7282 ± 3231
10 μM triclocarban	177,714 ± 19,834**	66,057 ± 14,675*

*p < 0.05 and **p < 0.01 versus the control cultures

### Triclocarban Inhibited the Histone Deacetylase, Sirtuin, and DNA Methyltransferase Activities But Did Not Affect the Histone Acetyltransferase Activity in Neocortical Cells

#### HDAC Activity

**Fig. 9:**
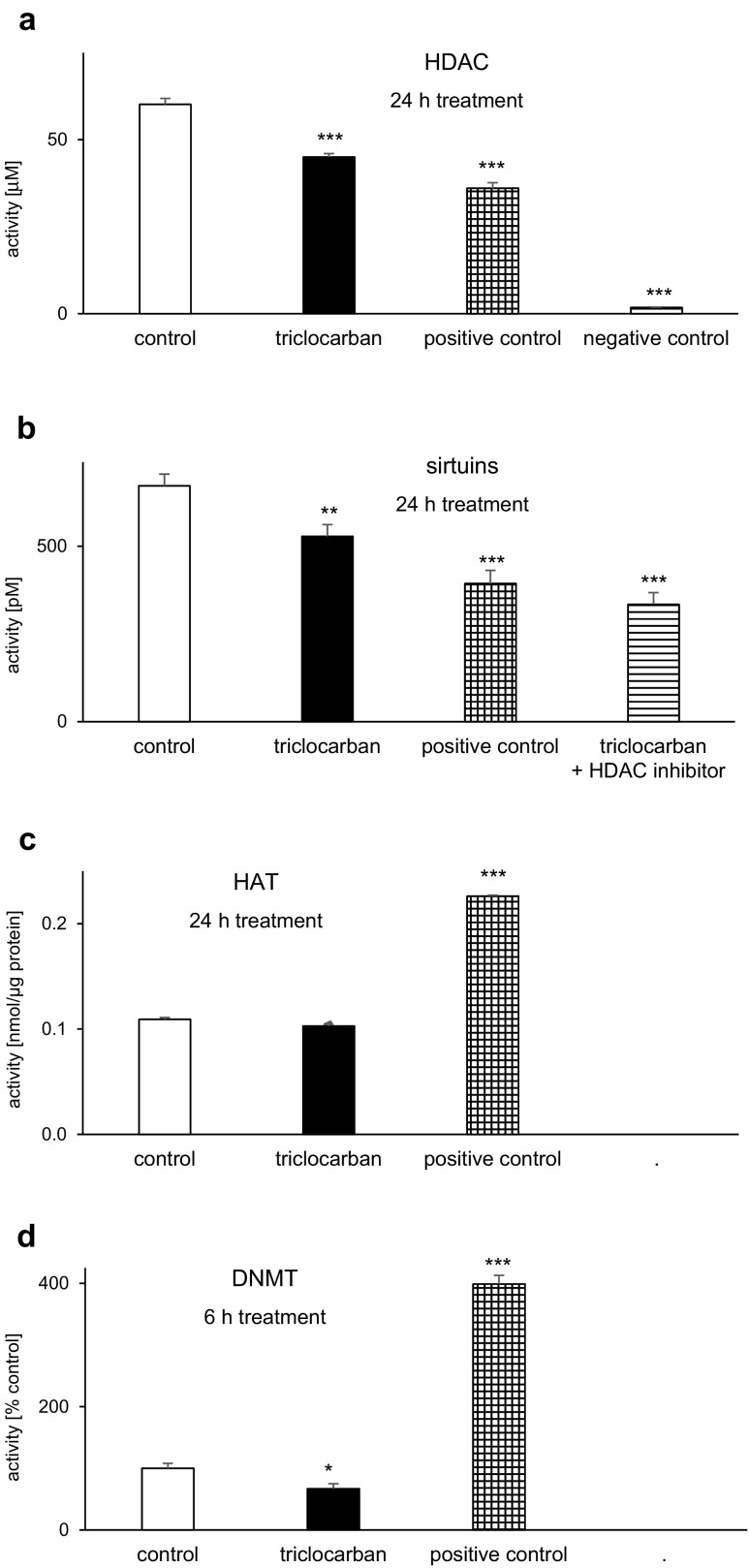
Triclocarban (10 μM) inhibited the histone deacetylase (HDAC; **a**), sirtuin (**b**), and DNA methyltransferase (DNMT; **d**) activities but did not affect histone acetyltransferase activity (HAT; **c**) in neocortical cells. The primary neocortical cultures were treated with triclocarban for 24 h, except for DNMT activity that was measured after 6-h treatment. A positive control contained HeLa cells for HDAC, undefined sample for sirtuins, an active nuclear extract for HAT and MU4 DNMT Enzyme Control for DNMT. A negative control had no cells, and the HDAC inhibitor has been used to inhibit non-sirtuin activities in the samples. The results were normalized to the absorbency in the vehicle-treated cells and expressed as μM, pM, nmol/μg protein, or a percentage of the control. Each bar represents the mean ± SEM. The number of replicates in each experiment ranged from 6 to 8, except for the measurements of sirtuin activity, whose number of replicates was 5. *p < 0.05, **p < 0.01, and ***p < 0.001 versus the control cultures

A 24-h treatment of neocortical cells with triclocarban (10 μM) decreased HDAC activity 25% compared with the control, i.e., from 60 to 45 μM (Fig. 9a). The level of triclocarban-affected HDAC activity was similar to the level observed in a positive control (HeLa cells) which was estimated to be 36 μM. A negative control (no cells) was determined to be approximately 2 μM.

#### Sirtuin Activity

In response to a 24-h exposure to 10 μM triclocarban, the sirtuin activity of mouse neocortical neurons decreased from 672 to 528 pM, which was equal to a 21% loss (Fig. 9b). An HDAC inhibitor inhibited both the control and triclocarban-affected sirtuin activities, which reached the values of 456 and 334 pM, respectively. Compared with the control, the values were 32 and 50% lower, respectively. In this study, a positive control (undefined sample provided by the manufacturer) displayed a level of 394 pM.

#### HAT Activity

In this study, a 24-h treatment with 10 μM triclocarban did not affect the HAT activity (Fig. 9c). A positive control (an active nuclear extract) displayed a level of 0.22 nmol/μg of protein sample.

#### DNMT Activity

Neocortical cells that were subjected to triclocarban (10 μM) for 6 h exhibited 33% lower DNMT activity than the control cells (Fig. 9d). A positive control (MU4 DNMT Enzyme Control) reached a value of approximately 400% compared with the control.

### Triclocarban Affected DNA Methylation

#### Triclocarban Caused Hypomethylation of Global DNA

**Fig. 10:**
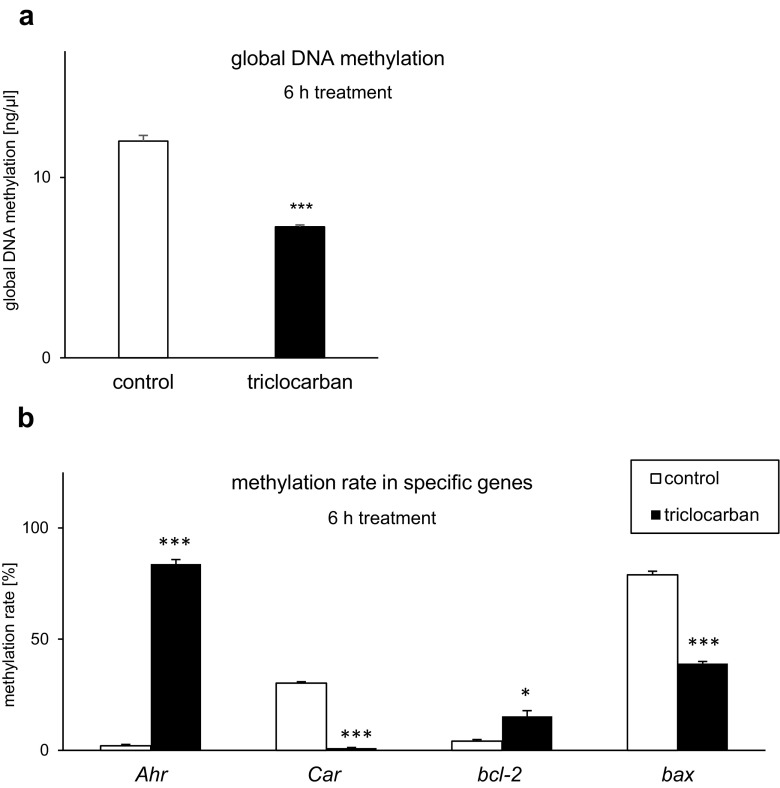
Global DNA hypomethylation and altered DNA methylation of specific genes in neocortical cells in primary cultures that were exposed to triclocarban (10 μM) for 6 h. Global DNA methylation (**a**) and the methylation rate estimated in specific genes, i.e., *Ahr*, *Car*, *bcl2*, and *bax* (**b**). Each bar represents the mean ± SEM. The number of replicates in each experiment ranged from 6 to 8. *p < 0.05 and ***p < 0.001 versus the control samples

In the control cells, the global DNA methylation reached a value of 12 ng/μl (Fig. 10a). After the treatment with triclocarban (10 μM), the global DNA methylation was reduced by approximately 5 ng/μl, i.e., 40%. Interestingly, the effect was already observed at 6 h after the initial treatment.

#### Triclocarban Altered DNA Methylation in Specific Genes

To support our data on triclocarban-evoked hypomethylation of global DNA, we analyzed the methylation statuses of specific genes (i.e., the receptor genes *Ahr* and *Car* and apoptosis-related genes *bcl2* and *bax*) in the mouse neocortical cells.

A 6-h exposure of the cells to triclocarban (10 μM) led to an approximately 80% increase in the DNA methylation of *Ahr* and 10% increase in the DNA methylation of *bcl2* (Fig. 10b). In contrast to the *Ahr* and *bcl2* genes, triclocarban reduced the DNA methylation of *Car* and *bax* by 29-39%. In all genes affected by triclocarban, the levels of unmethylated DNA were inversely correlated with the levels of methylated DNA.

### Triclocarban Inhibited Sumoylation

**Fig. 11:**
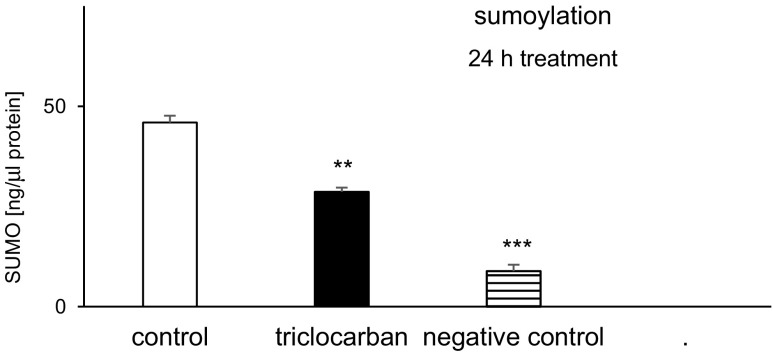
Triclocarban (10 μM) inhibited sumoylation in neocortical cells in the primary cultures, examined 24 h after the initial treatment. The results were normalized to the absorbency in the vehicle-treated cells and expressed as pM. Each bar represents the mean ± SEM. The number of replicates in each experiment ranged from 6 to 8. **p < 0.01 and ***p < 0.001 versus the control cultures

In the control neocortical cultures, the sumoylation level was estimated at 46 ng/μl of protein (Fig. 11). Treatment with triclocarban (10 μM) for 6 h reduced it by approximately 40%. The negative control’s sumoylation (no cells) remained at the level of 9 ng.

## Discussion

Following a concern related to common exposures to triclocarban that was recently verbalized as the so-called *The Florence Statement on Triclosan and Triclocarban* documents [43], we demonstrated that triclocarban at environmentally relevant concentrations induced apoptosis in mouse embryonic neurons as indicated by a substantial loss of mitochondrial membrane potential, a decrease in BCL2/BAX ratio, and apoptotic fragmentation of cell nuclei. These processes were accompanied by ROS formation, LDH release, and neuronal cell death. Our results are in accordance with cytotoxic effect of triclocarban used in combination with hydrogen peroxide in rat thymocytes [8]. The results are also in line with the lethality of rat pups that were fed by lactating females exposed to triclocarban [6]. In the present study, the effects of triclocarban were dose and time dependent, and the lowest effective concentration appeared to be 10 μM.

The tested concentrations of triclocarban ranged from 0.1 to 100 μM, with the most frequently used 10 μM triclocarban, i.e., 3155.8 μg/l. This concentration of triclocarban is environmentally relevant because the chemical has been found in human tissues at similar doses. For example, the exposure of Chinese population living in Beijing and Sichuan Province to triclocarban has been estimated at the levels of approximately 6.5 ± 27 μg/g creatinine (urine), 571 ± 1255 μg/kg (fingernails), and 259 ± 770 μg/kg (toenails). The maximal concentrations of triclocarban in urine, fingernails, and toenails reached the values of about 278 μg/g creatinine, 9006 μg/kg, and 9598 μg/kg, respectively [27]. Human samples of third trimester maternal urine (6–9th months) contained even 5.8 μg/g creatinine [28]. In healthy volunteers, the mean soap consumption was estimated at 11.7 ± 2.6 g (70 ± 15 mg triclocarban) that corresponds to an average a maximal topical dose of 1 mg/kg bodyweight equal to 40 mg/m^2^ body surface area [26].

In the present study, the use of selective antagonists (α-naphthoflavone, CH223191, and CINPA) and specific siRNAs, which was followed by the demonstration of co-localization of the aryl hydrocarbon receptor (AHR) and constitutive androstane receptor (CAR) in mouse neurons, points to the involvement of the AHR and CAR in triclocarban-induced apoptosis and neurotoxicity. There is no relevant publication to compare our results with. Previously, we showed that a selective AHR agonist, β-naphthoflavone, was able to initiate apoptosis in mouse neurons at an early developmental stage [44]. We also provided evidence for an essential role of AHR signaling in the propagation of deleterious effects of the pesticide DDT, triclosan, and hypoxia [16–18] and a participation of CAR in nonylphenol-induced apoptosis and neurotoxicity [22]. Recently, Du et al. [23] showed that AHR and CAR play roles in di-(2-ethylhexyl)-phthalate-induced cerebellar toxicity in quails. In the present study, triclocarban-stimulated functioning of the receptors was correlated with an increase in their protein levels after a prolonged 24-h exposure and a disruption of neurite outgrowth in neocortical neurons.

In addition to an induction of apoptosis and neurotoxicity, triclocarban altered the epigenetic status of mouse neurons evidenced by the impaired activities of HDAC, sirtuins, and DNMT as well as by the hypomethylation of global DNA. This effect could explain an adverse birth outcome, such as decreased gestational age at birth in human neonates who were prenatally exposed to triclocarban [28]. Previously, we demonstrated that the pesticide DDT and its metabolite DDE caused global DNA hypomethylation, both in the brains of adolescent mice, which were prenatally exposed to the pesticide, and in primary neurons in cultures that were treated with the chemical for several hours [38, 39]. Histone hypoacetylation induced by histone deacetylase activities is associated with gene silencing, whereas the hypomethylation of the DNA is linked to gene opening. Surprisingly, DNA methylation may predict increased instead of decreased gene expression that depends on CpG site, gene, and tissue specificity. Dense CpG promoters tend to be unmethylated, whereas low CpG promoters tend to be methylated. Furthermore, many hypomethylated dense CpG promoters are transcriptionally inactive, and hypermethylated low CpG promoters are required for activation of some tissue specific genes [45]. In the present study, the impaired activities of histone deacetylases and DNMTs could explain the decrease in global DNA methylation in response to triclocarban. We postulate that the triclocarban-evoked alteration of epigenetic status supports the synthesis of pro-apoptotic proteins and xenobiotic receptors, including AHR and CAR.

In the present study, a 24-h treatment with triclocarban enhanced the protein levels of the receptors which was paralleled by the hypomethylation of the *Car* and hypermethylation of the *Ahr* genes. The *Car* hypomethylation is in line with global DNA hypomethylation and explains the increased mRNA (3 and 6 h) and protein (24 h) levels of the receptor in response to triclocarban. The *Ahr* hypermethylation could reflect reduced *Ahr* mRNA expression but not increased AHR protein level in the cells which were exposed to triclocarban for 24 h. Nonetheless, the *Ahr* hypermethylation could correspond to lowered AHR protein levels after 3- and 6-h exposures to triclocarban that is likely related to proteasomal degradation of activated AHR. We hypothesize that the triclocarban-induced apoptosis in mouse neurons and the disruption of the epigenetic status involve both AHR- and CAR-mediated effects; however, the expression of the receptors is regulated by different means.

The AHR is known to interact with the estrogen receptor-mediated intracellular pathways, including receptor-regulated proteasomal degradation [46]. Moreover, the AHR has been found to degrade in the presence of high levels of ROS [47, 48]. Taking these into account, one may assume that in this study, the lack of correlation between *Ahr* methylation and AHR protein level at 24 h of exposure is partially due to triclocarban-evoked ROS formation that affects both the epigenetic status and protein expression of the receptor. With regard to CAR, its pattern of DNA methylation is similar to the pattern of *bax*, which supports a pro-apoptotic nature of CAR-mediated signaling activated by triclocarban in mouse neurons. Interestingly, according to our data, the *bax* gene hypomethylation parallels the enhanced BAX protein expression, whereas the *bcl2* gene hypermethylation is in line with reduced BCL2 protein expression.

According to our data, the treatment of mouse neurons with triclocarban inhibited posttranslational protein modifications in terms of sumoylation. This effect of triclocarban could be directly linked to an impairment of protective capacity of sumoylation, since increasing global sumoylation has been postulated to be a strategy for protecting the brain against ischemic damage [49] and HDAC1 sumoylation has been recognized as a naturally occurring defense mechanism protecting against Aβ toxicity [50]. Furthermore, sumoylation of AHR enhanced its stability and repressed the transactivation activity of the receptor [51]. Because a disruption of sumoylation in the developing brain appeared to be lethal, the process seems to exert a central role during embryonic and postnatal development [52]. We postulate that the triclocarban-evoked apoptosis and neurotoxicity involve an inhibition of sumoylation. Consequently, the impaired sumoylation could destabilize the AHR and/or CAR proteins and stimulate their transcriptional activities in mammalian neurons.

## Conclusions

In summary, our study demonstrated for the first time that the triclocarban-induced apoptosis of embryonic neuronal cells is a caspase-3- and BCL2/BAX-dependent process that involves the activation of AHR- and CAR-mediated signaling. The triclocarban-induced stimulation of the AHR and CAR signaling pathways was verified with the use of selective antagonists and specific siRNAs, which was supported by measurements of mRNA and protein expression and the demonstration of the co-localization of the receptors in mouse neurons. In addition, the triclocarban-induced global DNA hypomethylation correlated with the hypomethylation of the *Car* and *bax* genes but was inversely correlated with the hypermethylated *Ahr* and *bcl2* genes. In conclusion, this study demonstrates that the exposure of mouse neocortical cells to triclocarban, used at environmentally relevant concentrations, induces AHR- and CAR-mediated apoptosis, disrupts the epigenetic status of the neuronal cells, and inhibits posttranslational protein modifications in terms of sumoylation which may substantiate a fetal basis of the adult onset of neurological diseases.

## Abbreviations

AHR: Aryl hydrocarbon receptor
AM: Acetoxymethyl
ANOVA: Analysis of variance
ARNT: Aryl hydrocarbon receptor nuclear translocator
BSA: Bovine serum albumin
CAR: Constitutive androstane receptor
CH223191: 1-Methyl-*N*-[2-methyl-4-[2-(2-methylphenyl) diazenyl]phenyl-1*H*-pyrazole-5-carboxamide; AHR antagonist
CINPA: Ethyl [5-[(diethylamino)acetyl]-10,11-dihydro-5*H*-dibenz[*b*,*f*]azepin-3-yl]carbamate; CAR antagonist
DIV: Days in vitro
DMSO: Dimethyl sulfoxide
DNMT: DNA methyltransferase
ELISA: Enzyme-linked immunosorbent assay
HAT: Histone acetyltransferase
HDAC: Histone deacetylase
*Hprt*: Hypoxanthine phosphoribosyltransferase coding gene
JC-1: (5′,6,6′-Tetrachloro-1,1′,3,3′-tetraethylbenzimidazolylcarbocyanine iodide); the membrane-permeant dye that is widely used in apoptosis studies
LDH: Lactate dehydrogenase
qPCR: Quantitative polymerase chain reaction
ROS: Reactive oxygen species
RT: Reverse transcription
SUMO: Small ubiquitin-like modifier
